# Associations between affective distress, alcohol use, and cognitive flexibility: moderating effects of COMT Val158Met genotype

**DOI:** 10.3389/fpubh.2026.1901554

**Published:** 2026-09-01

**Authors:** Cody A. Mashburn, Emily M. Clarke, Sharon M. Pearcey, Emily M. Pogue, Bilal Saleem, Erica D. Holliday

**Affiliations:** 1 Department of Psychological Science, Neurobiological Examination of Addiction, Recovery, and Related Disorders, Radow College of Humanities and Social Sciences. Kennesaw State University, Kennesaw, GA, United States; 2 Department of Molecular and Cellular Biology, College of Science and Math, Kennesaw State University, Kennesaw, GA, United States

**Keywords:** alcohol use, cognitive flexibility, COMT, emerging adulthood, prevention

## Abstract

**Background:**

Alcohol use is common during emerging adulthood and has been associated with impairments in executive functioning, including cognitive flexibility. Individual differences in affective distress, early life adversity, and genetic variation in dopaminergic signaling pathways may contribute to variability in cognitive flexibility among young adults. The present study examined the relationships among proximal affective distress, distal early life stress, alcohol use, and the functional COMT Val158Met polymorphism.

**Methods:**

Seventy-two college-aged adults completed measures of adverse childhood experiences (ACEs), affective distress, alcohol use, and cognitive flexibility. Cognitive flexibility was assessed using both behavioral measures from a Number-Letter task and self-reported cognitive flexibility using the Cognitive Flexibility Inventory (CFI). An exploratory factor analysis was conducted on measures of depression, anxiety, stress, affect, and state–trait anxiety to derive a latent affective distress factor. General linear models examined the effects of affective distress, drinking status, and COMT Val158Met genotype, on cognitive flexibility outcomes.

**Results:**

Higher affective distress was associated with lower scores on the CFI Control subscale, indicating reduced perceived behavioral control. This relationship was significantly stronger among individuals reporting alcohol use. Affective distress also interacted with drinking status to predict behavioral cognitive flexibility, such that greater distress was associated with poorer task-switching performance only with prior alcohol use. This interaction was further moderated by COMT genotype. Specifically, Val/Val individuals showed greater reductions in cognitive flexibility conditions of elevated affective distress and alcohol use, whereas Met-carriers showed relatively stable performance across conditions. ACEs were positively associated with affective distress but did not predict cognitive flexibility outcomes.

**Conclusion:**

Proximal affective distress, but not early life adversity, emerged as a key correlate of both perceived and behavioral cognitive flexibility in emerging adults, particularly among individuals reporting alcohol use. COMT genotype moderated these relationships, suggesting that dopaminergic variation may influence sensitivity to current affective and alcohol-related cognitive demands with limited impacts from early life stress. These findings highlight the importance of considering affective, behavioral, and biological factors jointly when examining cognitive flexibility and alcohol-related risk during emerging adulthood.

## 1 Introduction

Alcohol use is prevalent among young adults aged 18–25, with approximately 47% reporting alcohol consumption within the previous month (1). Rates are similarly high among full-time college students, a subset of whom engage in binge or high-intensity drinking. Alcohol use during the college years is particularly concerning because it has been consistently associated with poorer academic outcomes and impairments in executive functioning (2–4). Given the widespread nature of alcohol use within college settings, prevention efforts frequently target college campuses. However, these approaches often fail to account for individual differences in neurocognitive vulnerability that may contribute to problematic alcohol use.

Executive functioning (EF) is a neuropsychological construct encompassing higher-order cognitive processes that support planning, cognitive control, and the execution of goal-directed behavior, including inhibitory control, working memory, and cognitive flexibility (5). These processes allow individuals to evaluate outcomes, adjust behavior based on feedback, and guide decision-making in dynamic environments. EF undergoes substantial development throughout adolescence and emerging adulthood, corresponding with ongoing maturation of prefrontal cortical systems and reaching relative stabilization in young adulthood (2, 6). Importantly, EF is not only relevant for laboratory-based cognitive performance but also predicts real-world outcomes, including academic achievement, identity development, and adaptive coping strategies (7, 8). In contrast, executive dysfunction has been linked to maladaptive coping and increased engagement in alcohol and other substance use behaviors (9). Additionally, effective executive functioning may confer resilience by buffering stress-related impairments and reducing health-related complaints and is a relevant construct to study for prevention and intervention in alcohol use due to shorter durations of lifetime drinking.

Cognitive flexibility, the ability to adapt strategies to guide goal-directed behavior, is a domain of executive functioning particularly vulnerable disruption by environmental insults during adolescence and emerging adulthood. Alcohol use, particularly when initiated during adolescence, is also associated with impairments in cognitive flexibility (10, 11). In community samples, higher levels of alcohol consumption were linked to poorer performance on measures including the Wisconsin Card Sorting Task and the Trail Making Test, indicating deficits in behavioral flexibility beyond self-report indices (12). Similarly, adolescent alcohol use has been shown to impair performance on Trail Making tasks in older but not younger adolescents (2), and to disrupt reversal learning while sparing initial acquisition (13). These age-dependent effects likely reflect differential vulnerability of prefrontal cortical systems during development. Importantly, such deficits may not be immediately apparent and may instead emerge following maturation of frontal circuitry (14).

While alcohol use represents one environmental factor associated with poorer cognitive flexibility, psychological stressors may exert similar or interacting effects. Indeed, depression, anxiety, and stress have each been linked to deficits in executive functioning, particularly cognitive flexibility. Patients diagnosed with major depressive disorder (MDD) demonstrate larger switch costs and make more errors on task-switching paradigms (15, 16). Zheng et al. (17) extended these findings by demonstrating larger switch costs for both emotional and non-emotional stimuli, suggesting that impaired cognitive flexibility in MDD is global rather than context specific. Similarly, higher levels of trait anxiety have been associated with poorer cognitive flexibility, particularly on reversal learning tasks that require individuals to adapt behavior following changes in reinforcement contingencies. Individuals with elevated anxiety exhibit greater difficulty overcoming previously learned response biases despite intact learning of task contingencies, suggesting that anxiety primarily disrupts the executive control processes underlying flexible behavior rather than learning itself (18). Collectively, these findings suggest that multiple dimensions of psychological distress may interfere with the executive processes needed for appropriate adaptive behaviors. Perceived stress has likewise been associated with reduced cognitive flexibility, with individuals reporting higher stress exhibiting poorer performance on set-shifting and adaptive decision-making tasks. Collectively, these findings suggest that multiple dimensions of negative affect may interfere with the executive processes necessary for flexible, goal-directed behavior during adolescence and emerging adulthood.

While affective distress reflects current psychological burden, developmental stress exposure may represent an additional pathway through which vulnerability to cognitive dysfunction and substance use emerges. Adverse childhood experiences (ACEs) represent distal cumulative exposure to early-life adversity and have been associated with alterations in stress responsivity, increased substance use risk, and differences in executive functioning (19). For example, greater ACE exposure has been linked to increased risk for substance use, whereas greater self-reported cognitive flexibility has been shown to attenuate this relationship in emerging adults (20). These findings further support the role of cognitive flexibility as a potential resilience factor across diverse forms of psychological adversity.

Stress, anxiety, and depression alter prefrontal cortical functioning through dopaminergic mechanisms thus genetic variation in dopamine regulation represents one plausible source of individual differences in vulnerability to affective distress-related impairments in executive functioning. A potential source of vulnerability involves genetic variation in systems associated with dopamine regulation and executive functioning, both of which have been implicated in alcohol use disorder (21). One candidate gene is catechol-O-methyltransferase (COMT), which encodes an enzyme responsible for the breakdown of dopamine in the prefrontal cortex (22). The Val158Met (rs4680) is a functional polymorphism with a valinine-methionine amino acid substitution that alters enzymatic activity, thereby influencing dopamine availability and prefrontal cognitive efficiency (23). The Val allele is associated with higher enzymatic activity and rapid dopamine degradation and associated with lower prefrontal dopamine signaling with compensatory upregulation of dopamine-1 (D1) receptors (24). In contrast, the met allele produces lowered enzymatic activity resulting in greater dopamine availability (25). Optimal prefrontal dopamine signaling follows an inverted U-shaped curve such that the cognitive and affective consequences of these allelic differences are not straightforward (23). Although met allele carriers have demonstrated enhanced performance in attentional tasks and cognitive stability (26–28) during low stress but become impaired during mild stress. Conversely, the val allele has been associated with reduced executive functioning in some conditions which is augmented by aging, but may confer advantages when dopamine signaling is elevated (29). Importantly, these allele-related differences have also been implicated in affective phenotypes, with COMT variation contributing to individual differences in anxiety (30), depressive symptoms (31), and stress responses (32).

Given that cognitive flexibility depends on intact PFC regulation and is also disrupted by depression, anxiety, and stress, COMT related differences in dopamine regulation may influence the extent to which affective distress translates into executive dysfunction. To clarify these interactions, the present study examined how affective distress, alcohol use, and genetic variation in dopaminergic and synaptic signaling pathways relate to cognitive flexibility in college-aged adults. Affective distress was operationalized as a latent factor reflecting shared variance across symptoms of depression, anxiety, and stress, representing a proximal measure of participants’ current affective state. In contrast, early life adversity was conceptualized as a distal developmental stressor reflecting exposure to adverse experiences during childhood and was examined as a grouping variable (low, intermediate, and high adversity). Alcohol use was examined as a grouping variable reflecting drinking status, i.e., alcohol users and non-users. Cognitive flexibility was assessed using both performance-based measures of task switching and self-report scales. Finally, we tested whether variation in COMT Val158Met moderated associations between affective distress, alcohol use, and cognitive flexibility outcomes.

To our knowledge, this is the first preliminary investigation to integrate these specific variables in a human sample. We hypothesized that higher affective distress, higher instances of ACES, and reported alcohol use would be associated with poorer cognitive flexibility across both behavioral and self-report measures. We further expected that these relationships would vary as a function of COMT genotype, with Val158Met genotype moderating the associations between affective distress, alcohol use, and cognitive flexibility. Given evidence that the cognitive effects of COMT are context dependent, we anticipated allele-specific patterns of vulnerability rather than uniform deficits across genotypes.

## 2 Methods

### 2.1 Participants and procedures

A total of 72 undergraduate students (see Table 1 for group sizes and participant demographics) were recruited through the university SONA research participation system as part of a larger ongoing study examining genetic, affective, and cognitive factors associated with substance use risk in emerging adults. The present study specifically focused on affective measures, ACEs, and cognitive flexibility, which was measured via behavioral performance on the Number–Letter Task and via self-report using the Cognitive Flexibility Inventory (33). All study procedures were approved by the university Institutional Review Board (IRB), and all participants provided informed consent prior to participation. Following consent, participants completed a series of demographic questionnaires and self-report measures assessing psychological functioning, adverse childhood experiences, and alcohol use behaviors. Participants then provided saliva samples for genetic analyses, followed by completion of computerized cognitive tasks assessing cognitive flexibility and executive functioning.

**Table 1:** Sample demographics.

Characteristic	Overall (*n* = 72)	Alcohol use status	
		Any prior use (*n* = 41)	No prior use (*n* = 31)
Genotype			
Val/Val	29	15	14
Met/Met	16	10	6
Val/Met	27	16	11
Gender			
Male	23	13	10
Female	49	28	21
Race			
Asian	5	2	3
Black/African American	15	6	9
White	39	27	12
Multiple	10	5	5
Unknown	3	1	2
Age, Mean (SD)	18.65 (1.05)	18.66 (1.09)	18.65 (1.02)

*N* = 72.

Saliva samples were collected using the passive drool method into sterile cryogenic collection tubes. Samples were initially stored at −20 °C and subsequently transferred to −80 °C prior to DNA extraction.

### 2.2 Surveys

Participants completed a series of standardized self-report measures assessing affective functioning, stress, alcohol use, adverse childhood experiences, and cognitive flexibility. Adverse childhood experiences were assessed using the Adverse Childhood Experiences (ACEs) questionnaire (19). ACES scores were grouped by risk for developing toxic stress symptoms; “Low Risk” participants report no adverse events, “Intermediate Risk” participants reported at least three adverse events, and “High Risk” participants reported at least 4 symptoms. Symptoms of depression, anxiety, and stress were measured using the Depression Anxiety Stress Scales-21 (DASS-21), which includes separate subscales assessing depressive symptoms, anxiety symptoms, and stress-related symptoms (34). Positive and negative affect were assessed using the Positive and Negative Affect Schedule (PANAS), including both positive affect and negative affect subscales (35). State and trait anxiety were measured using the State–Trait Anxiety Inventory (STAI), which includes separate measures of transient anxiety symptoms (state anxiety) and stable anxiety-related tendencies (trait anxiety) (36). Higher scores on each measure reflected greater endorsement of the respective construct. Self-reported cognitive flexibility was assessed using the Cognitive Flexibility Inventory, which has two subscales (33). The first, Control, indicates the degree to which participants believe that their behavior is adaptable, particularly to difficult circumstances. The second, Alternatives, concerns the number of alternative explanations and solutions participants can generate for difficult circumstances. Alcohol use status was determined by the Alcohol Use Disorders Identification Test (AUDIT) (37). Because our sample exhibited a restricted range of clinical alcohol severity with only three participants meeting the established cutoff for hazardous or harmful consumption (AUDIT score ≥ 8), the variable was dichotomized to evaluate the presence or absence of any alcohol consumption. The ‘“No prior alcohol use” group was defined strictly by an AUDIT score of 0 (*n* = 31; *M*_AUDIT_ = 0; *SD*_AUDIT_ = 0), while the “Alcohol use” group included any participant reporting alcohol consumption with an AUDIT score greater than 0 (*n* = 41; *M*_AUDIT_ = 3.415; *SD*_AUDIT_ = 3.521). This classification reflects *any* history of drinking rather than problematic or hazardous use. To maintain consistency with our analytic approach and to emphasize alcohol-use status *vs*. severity, relationships with raw AUDIT scores are omitted from our analyses.

### 2.3 Number-letter task

Cognitive flexibility was assessed behaviorally using the Number–Letter Task administered via Millisecond Inquisit software (38). This task is a set-shifting paradigm in which participants categorize number–letter pairs based on rule instructions that vary according to stimulus location on the screen (38). On each trial, a number–letter pair appeared in one of four screen quadrants. When the stimulus appeared in the upper quadrants, participants indicated whether the number was odd or even. When the stimulus appeared in the lower quadrants, participants indicated whether the letter was a vowel or consonant. Responses were made using designated keyboard keys corresponding to each rule.

The task included 128 experimental trials, consisting of 64 switch trials and 64 non-switch trials. Switch trials required participants to alternate between task rules based on stimulus location. Within switch trials, conditions were balanced across number and letter judgments, with equal representation of consonant/vowel and odd/even classifications, as well as congruent and incongruent trials. Congruent trials were defined as those in which response mappings between target and competing stimulus dimensions were aligned, whereas incongruent trials required competing response selections across dimensions.

Reaction time and accuracy were recorded on each trial and combined to form Linear Integrated Speed Accuracy scores (LISAs) using the following formula:

$$LISA_{ij}=CRT_{ij}+PE_{ij}\cdot \frac{S_{CRT_{j}}}{S_{PE_{j}}}$$

Where j indicates subject, i indicates condition (switch vs. non-switch), CRT indicates (average) correct reaction time, PE indicates the proportion of erroneous responses, and S indicates standard deviation (39, 40). Essentially, the LISA adds a weighted penalty to reaction times for inaccurate responding. Vandierendonck has shown that the LISA is among the best integrative measures for accounting for speed-accuracy interactions.

Cognitive flexibility in the Number-Letter Task was indexed by differences between LISA scores calculated on switch and non-switch trials, with greater switch costs (slower reaction time and/or reduced accuracy) reflecting reduced task-switching efficiency.

### 2.4 SNP genotyping by quantitative PCR

Genomic DNA was extracted using the Quick-DNA miniprep kit (Zymo Research, Irvine, CA) or the QIAmp DNA Mini Kit (Qiagen, Hilden, Germany) according to the manufacturers’ protocols. DNA concentration and purity were assessed prior to genotyping using spectrophotometric analysis. To minimize potential batch effects, DNA samples were normalized to a uniform concentration of 1 ng/μL prior to genotyping.

Single nucleotide polymorphism (SNP) genotyping was performed using quantitative polymerase chain reaction (qPCR) with allele-specific TaqMan SNP Genotyping Assays (Applied Biosystems, Foster City, CA, USA) on a BioRad CFX Opus 96 Real-Time PCR System. Target SNP *COMT* rs4680 20X (Assay ID: C__25746809_50; Cat. No. 4362691). Assays were performed using the TaqMan Genotyping Master Mix (Cat. No. 4444557). Reactions were prepared in a total volume of 25 μL containing master mix (12.5uL), TaqMan SNP Genotyping Assay (1.25uL), template DNA (10uL for 10 ng total DNA) and nuclease-free water according to manufacturer recommendations.

Thermal cycling conditions consisted of an initial denaturation step at 95C for 10 min, followed by 40 cycles of denaturation at 95C for 15 s and annealing and extension at 60C for 60 s. All samples were run in duplicate, and no-template controls were included in each run for quality assurance.

Genotype calls were determined using automated allele discrimination software associated with the qPCR platform (CFX Maestro) and were manually reviewed for accuracy. Samples with ambiguous amplification curves or failed reactions were repeated. There were three genotypic categories: homozygous methionine (Met/Met), homozygous valine (Val/Val), and heterozygous (Het). Due to the limited number of Met/Met homozygotes, Val/Met and Met/Met participants were combined into a Met-carrier group to improve statistical power, consistent with prior COMT Val158Met studies (41) Thus, primary genotype contrasts compared Val/Val homozygotes with individuals carrying at least one Met allele. Quality control procedures included assessment of call rates and consistency across replicate samples.

### 2.5 Statistical analysis

#### 2.5.1 Data preparation and dimension reduction

Prior to our primary analyses, we evaluated the distributional properties of all continuous measures to confirm they did not substantially deviate from normality. We assessed the scale reliability and internal consistency of all questionnaires and behavioral measures using McDonald’s omega ( ω ) or Spearman-Brown corrected split-half reliability, as appropriate (see Table 2).

**Table 2:** Overall and group-wise descriptive statistics and internal consistency estimates.

Group	Variable	*n*	Mean	SD	Range	Skew	Kurtosis	Internal consistency
Overall	ACES score	72	2.15	2.22	0–9	0.88	−0.09	0.75^ω^
	DASS-21 depression	72	8.36	8.16	0–36	1.55	2.35	0.90^ω^
	DASS-21 anxiety	72	9.83	8.77	0–42	1.28	1.98	0.87^ω^
	DASS-21 stress	72	13.31	8.33	0–36	0.47	0.11	0.88^ω^
	PANAS (positive)	72	33.26	8.25	15–50	−0.18	−0.48	0.89^ω^
	PANAS (negative)	72	21.40	7.26	10–42	0.72	0.12	0.85^ω^
	STAI state score	72	36.90	11.57	20–71	0.68	−0.01	0.94^ω^
	STAI trait score	72	45.29	12.57	20–74	0.03	−0.75	0.94^ω^
	NL accuracy cost	72	0.07	0.07	−0.02-0.30	1.41	2.01	0.49^SB^
	NL RT cost	72	580.78	177.86	263.80–1121.70	0.38	−0.11	0.74^SB^
	NL LISA cost	72	680.47	200.98	273.07–1151.62	0.11	−0.60	0.68^SB^
	CFI alternatives score	72	72.13	9.29	49–91	−0.24	−0.10	0.86^ω^
	CFI control score	72	30.86	7.7	8–47	−0.41	−0.09	0.82^ω^
No prior alcohol use × Val/Val	ACES score	14	1.71	1.98	0–5	0.48	−1.70	—
	DASS-21 depression	14	6.57	5.35	0–16	0.43	−1.14	—
	DASS-21 anxiety	14	7.29	6.01	0–18	0.35	−1.37	—
	DASS-21 stress	14	11.57	8.92	0–24	0.16	−1.59	—
	PANAS (positive)	14	35.50	6.90	24–29	0.34	−0.78	—
	PANAS (negative)	14	21.43	8.54	10–36	0.31	−1.17	—
	STAI state score	14	37.14	10.92	20–55	0.09	−1.30	—
	STAI trait score	14	44.57	12.09	22–58	−0.68	−1.07	—
	NL accuracy cost	14	0.05	0.06	−0.02 to 0.18	1.06	−0.23	—
	NL RT cost	14	627.68	236.79	327.53–1121.70	0.51	−0.86	—
	NL LISA cost	14	707.84	231.72	364.54–1151.62	0.23	−0.89	—
	CFI alternatives score	14	73.64	7.57	61–91	0.34	−0.10	—
	CFI control score	14	33.57	7.14	21–47	−0.06	−0.86	—
No prior alcohol use × Met-carriers	ACES score	17	1.47	1.74	0–6	1.19	0.44	—
	DASS-21 depression	17	8.24	10.51	0–36	1.42	0.75	—
	DASS-21 anxiety	17	9.88	11.71	0–42	1.43	1.25	—
	DASS-21 stress	17	11.18	9.93	0–36	1.14	0.40	—
	PANAS (positive)	17	33.71	9.93	16–48	−0.31	−1.21	—
	PANAS (negative)	17	20.06	6.94	10–35	0.64	−0.75	—
	STAI state score	17	35.47	14.01	20–63	0.57	−1.26	—
	STAI trait score	17	44.24	13.74	20–70	0.10	−1.18	—
	NL accuracy cost	17	0.06	0.08	−0.02 to 0.25	0.96	−0.38	—
	NL RT cost	17	587.40	178.69	286.98–832.45	−0.19	−1.33	—
	NL LISA cost	17	691.23	216.52	273.07–1092.59	−0.09	−0.83	—
	CFI alternatives score	17	72.24	10.67	51–89	−0.38	−0.86	—
	CFI control score	17	31.12	6.93	17–42	−0.09	−0.93	—
Alcohol use × Val/Val	ACES score	15	1.93	2.28	0–6	0.72	−1.18	—
	DASS-21 depression	15	7.20	7.40	0–24	1.14	0.04	—
	DASS-21 anxiety	15	9.87	9.02	0–30	0.56	−0.82	—
	DASS-21 stress	15	13.20	10.05	0–36	0.60	−0.50	—
	PANAS (positive)	15	31.73	8.87	17–46	−0.11	−1.39	—
	PANAS (negative)	15	20.07	7.76	12–42	1.45	1.65	—
	STAI state score	15	33.40	9.15	20–56	0.63	0.15	—
	STAI trait score	15	42.80	14.34	27–71	0.50	−1.19	—
	NL accuracy cost	15	0.07	0.07	−0.02 to 0.28	1.41	1.71	—
	NL RT cost	15	625.28	175.82	318.67–837.05	−0.47	−1.33	—
	NL LISA cost	15	732.11	214.68	397.95–1023.84	−0.35	−1.59	—
	CFI alternatives score	15	73.47	9.84	53–90	−0.23	−0.46	—
	CFI control Score	15	30.47	8.73	16–42	−0.40	−1.43	—
Alcohol use × Met-carriers	ACES score	26	2.96	2.46	0–9	0.57	−0.60	—
	DASS-21 depression	26	10.08	8.19	0–34	1.31	1.52	—
	DASS-21 anxiety	26	11.15	7.80	0–30	0.86	−0.10	—
	DASS-21 stress	26	15.69	5.05	6–26	0.07	−0.75	—
	PANAS (positive)	26	32.65	7.53	15–50	−0.09	−0.20	—
	PANAS (negative)	26	23.04	6.48	12–39	0.53	−0.26	—
	STAI state score	26	39.73	11.33	24–71	0.91	0.30	—
	STAI trait score	26	47.81	11.21	30–74	0.17	−0.72	—
	NL accuracy cost	26	0.08	0.07	0.00–0.30	1.52	2.66	—
	NL RT cost	26	525.53	132.03	263.80–764.01	−0.03	−0.84	—
	NL LISA cost	26	628.90	155.56	299.81–920.05	−0.15	−0.89	—
	CFI alternatives score	26	70.46	9.10	49–85	−0.18	−0.57	—
	CFI control score	26	29.46	7.89	8–43	−0.51	0.10	—

ACES, Adverse Childhood Experiences; DASS, Depression, Anxiety, and Stress Scale; PANAS, Positive and Negative Affect Scale. STAI, State–Trait Anxiety Inventory; NL, Number Letter Task; RT, reaction time; LISA, Linear Integrated Speed-Accuracy Score; CFI, Cognitive Flexibility Inventory. ^ω^Indicates McDonald’s omega. ^SB^Indicates split-half reliability with the Spearman-Brown correction; split was randomly determined.

To reduce the dimensionality of our mental health metrics (DASS-21, STAI, and PANAS subscales), we subjected the questionnaires to an exploratory factor analysis (EFA) using maximum likelihood estimation. We determined the number of underlying factors to extract using a parallel analysis, a scree plot, and the Kaiser-Guttman rule. Based on these criteria, a single-factor solution was extracted (see Table 3). We calculated Bartlett factor scores from this solution to represent a singular “Affective Distress” index in all subsequent models.

**Table 3:** Standardized pattern matrix of EFA solution on mental health questionnaires.

Variable	Affective distress
DASS-21 depression	0.85
DASS-21 anxiety	0.76
DASS-21 stress	0.82
PANAS (positive)	−0.54
PANAS (negative)	0.82
STAI state score	0.85
STAI trait score	0.90

Factor extraction was performed via maximum likelihood estimation. DASS, Depression, Anxiety, and Stress Scale; PANAS, Positive and Negative Affect Scale. STAI, State–Trait Anxiety Inventory.

#### 2.5.2 Primary and *post-hoc* analyses

We first computed zero-order Pearson correlations to examine the bivariate relationships among our cognitive flexibility metrics, affective distress, and adverse childhood experiences (ACEs). To test our primary hypotheses regarding interactive effects on cognitive flexibility [measured via Number-Letter Task LISA switch costs, the Cognitive Flexibility Inventory (CFI) Control Score, and the CFI Alternatives Score, we conducted separate general linear models (GLMs) with Affective Distress and ACEs]. Affective distress and ACEs were modeled as separate variables due to their distinct temporal roles. Affective distress reflects participants’ current emotional state and proximal psychological demands at the time of testing (35), whereas ACEs capture distal exposure to childhood adversity and developmental stress (19). Separating these measures allowed examination of whether current affective burden versus early-life adversity differentially contributed to cognitive outcomes and moderated associations with alcohol use and COMT genotype. These models evaluated the interactive effects of drinking status, COMT genotype, and Affective Distress or ACEs. All GLMs were fitted using the aov_car() function from the *afex* R package (54), treating all categorical predictors as between-subjects factors. Affective distress factor scores served as a between-subject covariate.

To interpret significant interactions and evaluate simple slopes, we performed *post-hoc* conditional interaction contrasts and simple slopes analyses using the emmeans R package (42). We controlled the inflation of Type I error rate across multiple comparisons using Bonferroni adjustments. All statistical tests were evaluated at a family-wise significance threshold of α=.05 , where single omnibus effect decompositions (e.g., contrasts contributing to a main or interaction effect) constitute an effect “family.” For these *post-hoc* tests, p -values and confidence intervals were directly adjusted based on the number of comparisons ( k ) in that family (e.g., k=3 pairwise comparisons tested against an adjusted α=.05/3 , with $p_{adj}=\min\left(1,\;\;p\times k\right)$ ). We report unstandardized regression coefficients (B) and use generalized eta-squared ( $\eta_{G}^{2}$ ) as our measure of effect size. Primary model parameters report unadjusted 95% CIs, whereas adjusted values for *post-hoc* tests are indicated by the subscript “*adj*” (e.g., $95\%CI_{adj}$ ). For all Bonferroni adjustments, the number of corrected comparisons is noted in relevant footnotes and/or tables.

## 3 Results

### 3.1 Descriptives

Sample characteristics are presented in Table 1. Table 2 displays descriptive statistics for all questionnaires and behavioral measures, both for the whole sample and for each group in our design. Evaluating distributional properties indicated that measures do not substantially deviate from normality. Furthermore, all questionnaires displayed excellent internal consistency, with McDonald’s *ω* ranging from 0.75 to 0.94. Internal consistency of behavioral cognitive flexibility measures was lower, consistent with prior research (43, 44).

### 3.2 Questionnaires and dimension reduction

We subjected the mental health questionnaires to an exploratory factor analysis; the factor was extracted via maximum likelihood estimation. A principal components analysis revealed that a single component underlies scores across all DASS-21, STAI, and PANAS subscales, according to a scree plot, parallel analysis, and the Kaiser-Guttman rule. The final factor solution is presented in Table 3. Since more negative affect results in higher scores on this factor, we label this factor “Affective Distress.” From this solution, we obtained Bartlett factor scores to be used in subsequent analyses.

### 3.3 Cognitive flexibility and psychological distress measures

The relationships among measures of cognitive flexibility and affective distress are displayed in Table 4. A few things are notable. First, reporting more adverse childhood experiences is associated with higher levels of self-reported affective distress. Our primary behavioral index of cognitive flexibility is the LISA cost, an integrative measure combining reaction time and accuracy (39, 40). Unsurprisingly, both reaction time and accuracy switch costs correlated with LISA cost; no other measure correlated with behavioral cognitive flexibility metrics. Interestingly, both subscales of the Cognitive Flexibility Index correlated positively, but only the Control score was predicted by Affective Distress.

**Table 4:** Correlations among mental health risk factors and cognitive flexibility measures.

Variable	Affective distress	ACES score	NL accuracy cost	NL RT cost	NL LISA cost	CFI alternatives score	CFI control score
Affective distress	—	0.31^**^	**0.20**	**0.06**	**0.11**	**−0.18**	−0.66^***^
ACES score		—	**0.15**	**−0.07**	**0.01**	**−0.06**	**−0.19**
NL accuracy cost			—	**0.03**	0.35^**^	**−0.04**	**−0.13**
NL RT cost				—	0.93^***^	**0.00**	**−0.18**
NL LISA cost					—	**0.01**	**−0.21**
CFI alternatives score						—	0.41^***^
CFI control score							—

ACES, Adverse Childhood Experiences; NL, Number Letter Task; RT, reaction time; LISA, Linear Integrated Speed-Accuracy Score; CFI, Cognitive Flexibility Inventory. ***Significant at the *α* = 0.001 level. **Significant at the *α* = 0.01 level. *Significant at the *α* = 0.05 level. Bold values are not significant.

### 3.4 Interactive effects of COMT genotype, alcohol use, and affective distress on cognitive flexibility

#### 3.4.1 Number-letter task

Our first GLM tested interaction of COMT genotype, drinking status, and affective distress on Number Letter LISA cost. This analysis revealed a significant two-way interaction between drinking status and affective distress, *F*(1, 64) = 6.90, *p* = 0.011, η^2^_G_ = 0.097. Evaluating simple slopes (see Table 5) revealed that, among non-drinkers, there was a non-significant negative relationship between affective distress and LISA cost. Among prior alcohol use, there was a significant positive relationship between affective distress and LISA cost, indicating worse cognitive flexibility with greater distress.

**Table 5:** Simple slopes for the two-way interaction of alcohol use group and affective distress predicting number letter task LISA costs.

Alcohol group	Simple slope	SE	df	95% CI_adj_	*t*	*p_adj_*
No prior use (*n* = 31)	−41.76	32.84	64	[−117.13, 33.62]	−1.27	0.42
Prior use (*n* = 41)	76.74	30.91	64	[5.78, 147.69]	2.48	0.03^*^

*Significant at the *α* = 0.05 level. Both CIs and *p*-values have been adjusted via Bonferonni correction; *k* = 2.

**Figure 1:**
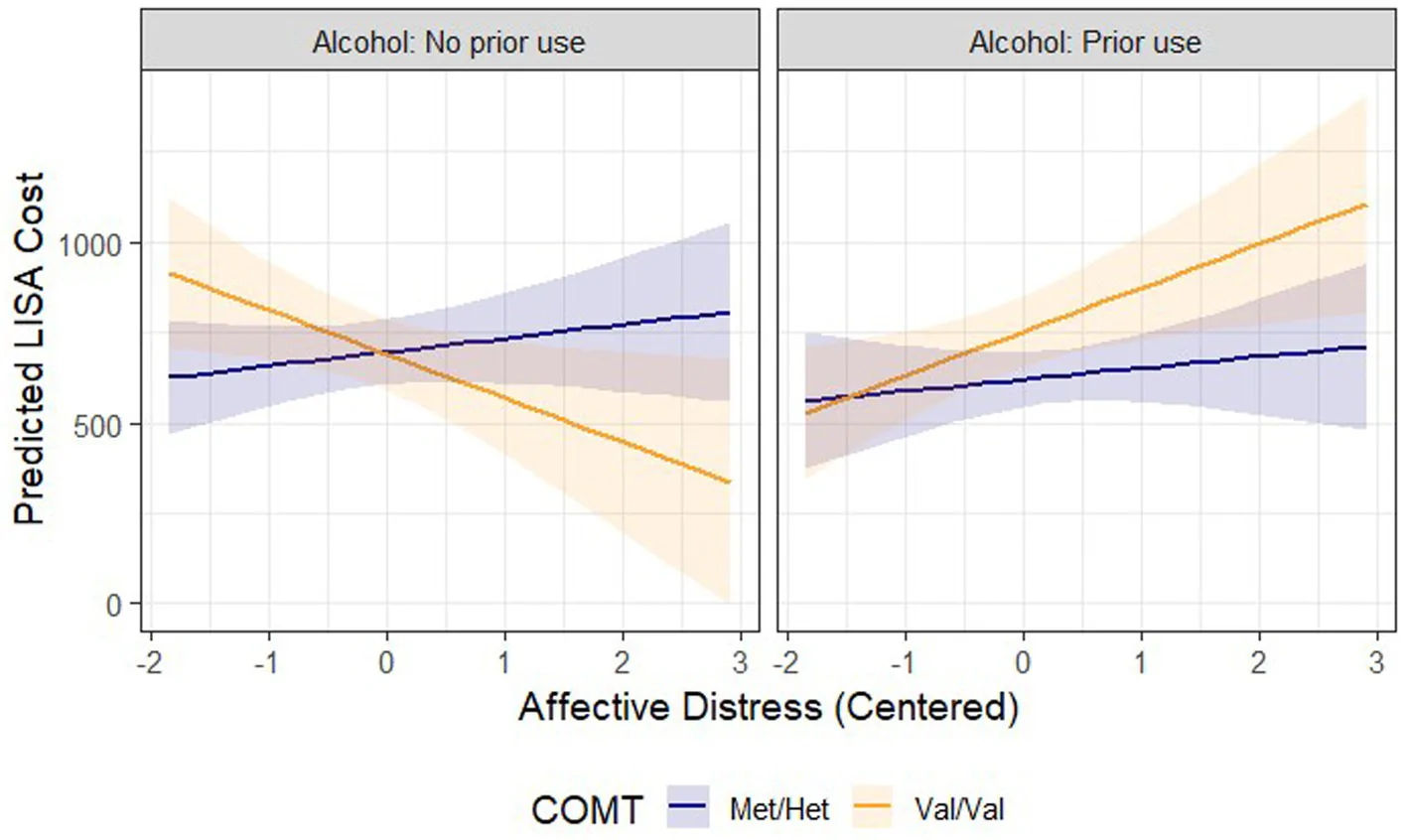
Effect of affective distress on LISA cost moderated by COMT genotype and alcohol use. Colored ribbons indicate 95% confidence intervals.

This two-way interaction was modulated by a significant three-way interaction, *F*(1, 64) = 7.66, *p* = 0.007, η^2^_G_ = 0.107. Conditional interaction contrasts and simple slopes comparing across levels of drinking status were conducted to assess this pattern. These revealed two primary findings. First, we found evidence that the two-way interaction between drinking status and affective distress differed by COMT genotype (Figure 1). For the Val/Val genotype, drinking combined with high affective distress predicted worse cognitive flexibility, while the opposite trend occurred among those with no prior alcohol use, *t*(64) = −3.44, *p* = 0.002, 95% CI [−405.64, −80.99]. No differences emerged for the Met-carriers genotype, *t*(64) = 0.113, *p* = 1.00, 95% CI [−122.18, 134.84]. Second, to gain a different (statistically equivalent) vantage on these data, we also assessed conditional simple slopes for each subgroup individually (see Table 6). The only group to show a significant relationship between affective distress and cognitive flexibility possessed both the Val/Val genotype and reported prior drinking behaviors. This trend indicates that those with the Val/Val genotype and who have engaged in drinking behaviors exhibit worse cognitive flexibility. No other trend was statistically significant.

**Table 6:** Simple slopes for the three-way interaction of COMT genotype, alcohol use group, affective distress predicting number letter task LISA costs.

Group	Simple slope	SE	df	95% CI_adj_	*t*	*p*_adj_
No prior alcohol × Val/Val (*n* = 14)	−121.70	53.57	64	[−259.40, 15.99]	−2.27	0.11
No prior alcohol × Met/Het (*n* = 17)	38.19	37.99	64	[−59.46, 135.84]	1.01	1.00
Prior alcohol × Val/Val (*n* = 15)	121.61	46.16	64	[2.96, 240.26]	2.63	0.04^*^
Prior alcohol × Met/Het (*n* = 26)	31.86	41.12	64	[−73.84, 137.57]	0.76	1.00

*Significant at the *α* = 0.05 level. Both CIs and *p*-values have been adjusted via Bonferonni correction. *k* = 4.

#### 3.4.2 Cognitive flexibility inventory control score

Our second GLM tested the interactions among COMT, drinking behavior, affective distress for predicting a different cognitive flexibility outcome, and the Control Score from the Cognitive Flexibility Inventory. This subscale assesses the tendency for one to believe that their behavior in the face of difficult circumstances is controllable, a prerequisite for adaptive responses to life stressors. Higher scores on this measure indicate a stronger belief in behavioral plasticity. Evaluating this model reveals two main results. First, consistent with zero-order correlations reported in Table 4, there was a significant main effect of Affective Distress, *F*(1, 64) = 49.760, *p* = < 0.001, η^2^_G_ = 0.437; those reporting greater distress also report less behavioral control, *B* = −4.81, 95% CI [−6.17, −3.45].

**Figure 2:**
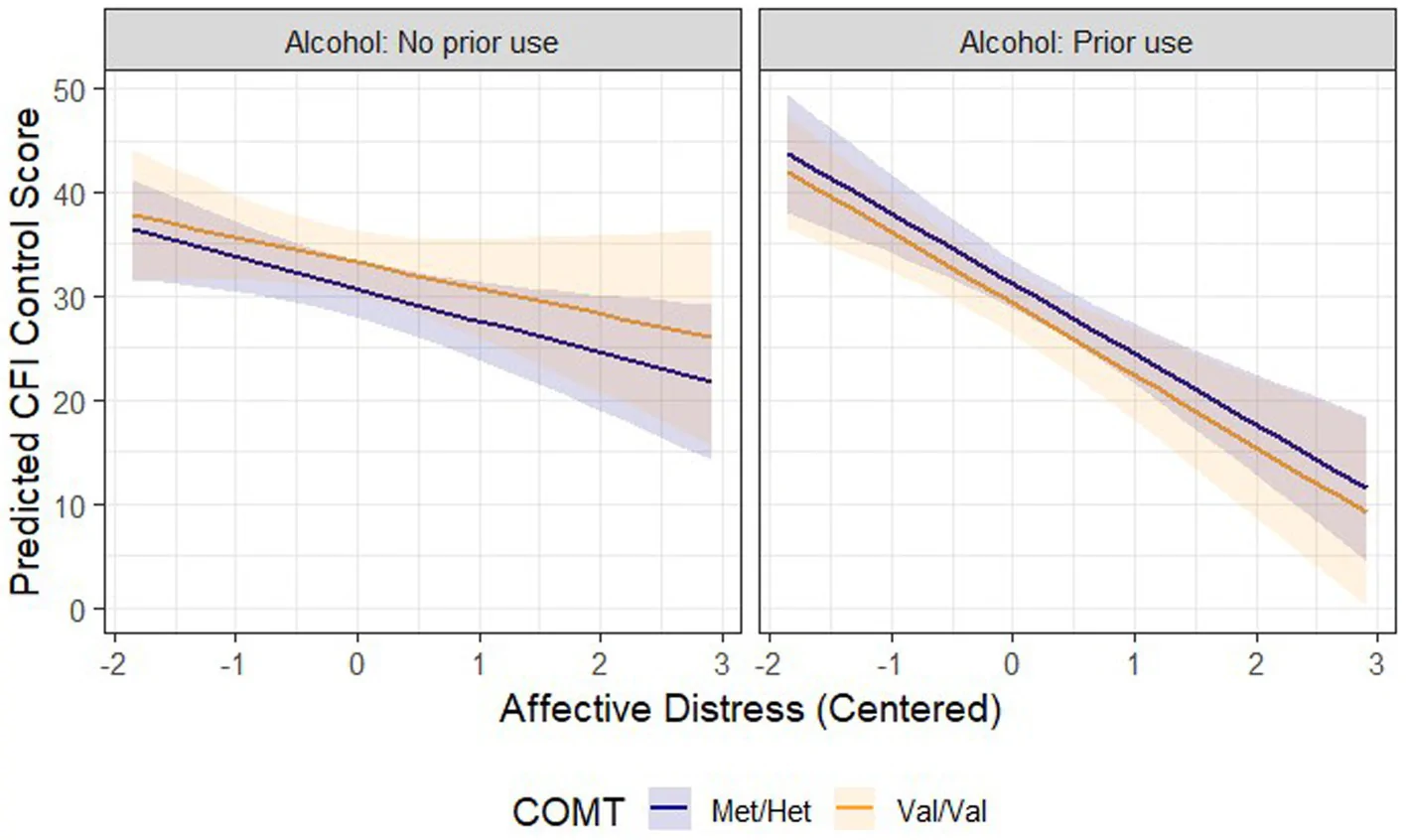
Effect of affective distress on CFI control score moderated by COMT genotype and alcohol use. Colored ribbons indicate 95% confidence intervals.

This main effect was moderated by a significant two-way interaction between affective distress and drinking status, *F*(1, 64) = 8.87, *p* = 0.004, η^2^_G_ = 0.07. Specifically, there was a stronger negative relationship between affective distress and perceived behavioral control among the alcohol use than among the no alcohol use (see Table 7 for simple slopes). Thus, while greater levels of affective distress predict lower self-reported control generally, the effect is especially acute for those reporting drinking behaviors (see Figure 2). No other effect was significant.

**Table 7:** Simple slopes for the two-way interaction of alcohol use group and affective distress predicting CFI control scores.

Alcohol group	Simple slope	SE	df	95% CI*_adj_*	*t*	*p_adj_*
No prior use (*n* = 31)	−2.78	0.99	64	[−5.06, −0.50]	−2.80	0.014^**^
Prior use (*n* = 41)	−6.84	0.93	64	[−8.99, −4.70]	−7.32	< 0.001^***^

**Significant at the *α* = 0.01 level; ***Significant at the *α* = 0.001 level. Both CIs and *p-*values have been adjusted via Bonferonni correction. *k* = 2.

#### 3.4.3 Cognitive flexibility inventory alternatives score

Our third statistical model substituted the CFI Control Score for the Alternatives Score. This metric reflects one’s propensity for generating alternative explanations and solutions to adverse conditions. No effect in this model approached statistical significance, largest *F*(1, 64) = 1.54, *p* = 0.219, η^2^_G_ = 0.022.

### 3.5 Adverse childhood experiences

**Figure 3:**
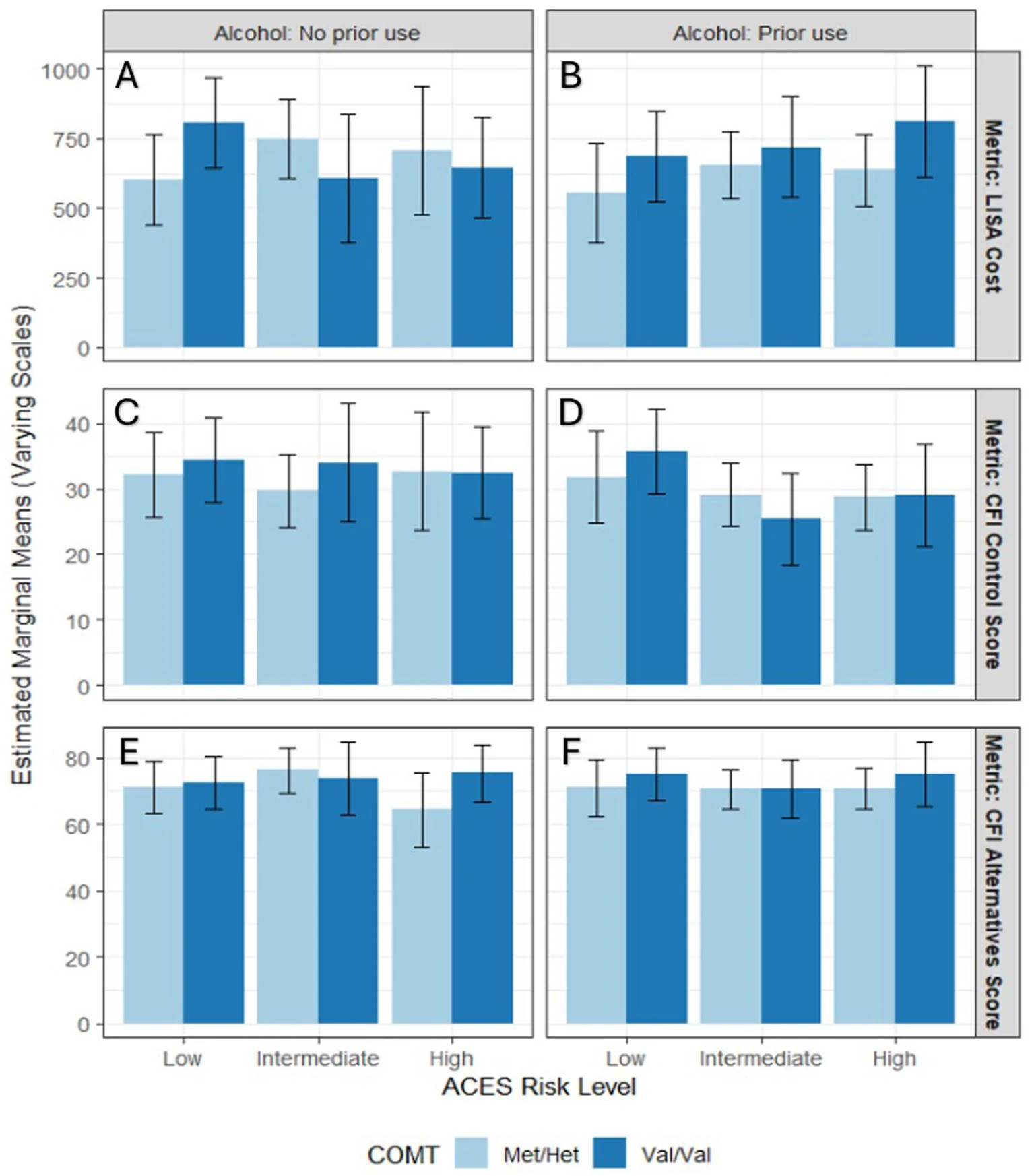
Cognitive flexibility metrics across ACES, drinking, and COMT genotype. **(A,B)** No difference in LISA cost between conditions. **(C,D)** No difference in CFI control across conditions. **(E,F)** No difference in CFI alternative scores across conditions. Error bars indicate 95% confidence intervals.

For our next set of analyses, we examined the effects of drinking, early life difficulty, and COMT genotype on cognitive flexibility. To this end, we substituted Toxic Stress Risk (as indicated by the number of adverse childhood experiences reported) for Affective Distress. Across LISA Costs, CFI Control and CFI Alternative Scores, no effects from the Adverse Childhood Experiences Model approached significance, largest *F*(1, 64) = 1.71, *p* = 0.196, η^2^_G_ = 0.025. See Figure 3 for a graphical depiction.

## 4 Discussion

The present study examined how affective distress, alcohol use, and genetic variation in dopaminergic signaling relate to cognitive flexibility in college-aged adults. Across models, affective distress emerged as a robust predictor of both perceived and behavioral aspects of cognitive flexibility. Specifically, higher affective distress was associated with lower perceived behavioral control and poorer performance on task-based measures of cognitive flexibility, particularly among individuals reporting alcohol use. In contrast, adverse childhood experiences (ACEs) were not significantly associated with cognitive flexibility outcomes. COMT genotype moderated several of these relationships, suggesting that dopaminergic variation may influence sensitivity to affective and alcohol-related cognitive demands.

Affective distress was strongly associated with lower scores on the Cognitive Flexibility Inventory Control subscale, indicating that individuals reporting higher levels of depression, anxiety, negative affect and stress also perceived themselves as having reduced control over managing difficult situations. This finding is consistent with literature linking elevated negative affect and internalizing symptoms to diminished perceived agency and reduced adaptive coping capacity (20) others have reported deficits in task switching as a clinically relevant endophenotype of depression (15). Together, this suggests a bidirectional relationship between affect and cognitive flexibility.

Importantly, this relationship was stronger among individuals reporting alcohol use, suggesting that alcohol consumption may exacerbate the association between affective distress and reduced perceived cognitive control. Rather than reflecting a classic locus of control construct, these findings are more consistent with the interpretation that affective distress shapes subjective evaluations of behavioral adaptability, particularly in the context of alcohol use. This pattern aligns with prior work demonstrating that alcohol use and negative affect are jointly associated with maladaptive cognitions during emerging adulthood (45, 46).

In addition to self-reported control, affective distress also predicted poorer performance on the Number-Letter task, but this effect was specific to individuals reporting alcohol use. This suggests that alcohol use may increase vulnerability to the negative cognitive effects of affective distress on behavioral flexibility, particularly in task-switching contexts. These findings are broadly consistent with evidence linking depression and negative mood states to altered executive functioning, including task-switching performance and neural markers of cognitive control (15, 47, 48). However, these studies report mixed effects of transient negative mood on cognitive flexibility, suggesting that acute negative affect may enhance certain aspects of task performance while impairing others depending on task demands. The present findings may therefore reflect a more sustained or trait-like form of affective distress, which appears more reliably associated with impaired cognitive flexibility, particularly in the presence of alcohol use.

COMT genotype moderated the relationship between affective distress, alcohol use, and cognitive flexibility performance. Specifically, individuals with the Val/Val genotype showed greater reductions in cognitive flexibility under conditions of elevated affective distress and alcohol use, whereas Met-carriers participants demonstrated relatively more stable performance across conditions. COMT influences prefrontal dopamine availability through enzymatic degradation, and variation in Val158Met has been associated with differences in executive functioning efficiency and cognitive stability. The Val allele is associated with greater COMT enzymatic activity and increased dopamine degradation in the prefrontal cortex, whereas the Met allele is associated with lower enzymatic activity and relatively greater dopaminergic availability. Previous work has reported enhanced cognitive flexibility among Val carriers in infants (49), healthy adults (50) and individuals with schizophrenia (51), while Met carriers have been associated with greater cognitive stability. The present findings are consistent with a moderation model in which dopaminergic efficiency shapes sensitivity to contextual and affective demands. In this framework, Val/Val individuals, characterized by higher COMT activity and reduced prefrontal dopamine availability, may be more vulnerable to disruptions in cognitive flexibility under conditions of combined affective distress and alcohol exposure. Importantly, these effects appear context-dependent rather than reflecting a uniform advantage or disadvantage of either genotype, suggesting that COMT-related differences emerge primarily under increased affective and behavioral load.

Together, these results suggest that affective distress is a central factor associated with both perceived and behavioral cognitive flexibility in emerging adulthood, particularly among individuals reporting alcohol use. Genetic variation in dopaminergic signaling, indexed by COMT Val158Met, further moderates these associations, indicating that biological sensitivity may influence how affective and behavioral risk factors translate into cognitive control differences. These findings highlight the importance of considering affective state and alcohol use jointly when examining cognitive flexibility, rather than treating these constructs as independent predictors. They also suggest that individual differences in dopaminergic regulation may contribute to variability in susceptibility to affect related cognitive control impairments.

From a public health perspective, these findings underscore affective distress as a potentially modifiable target for interventions aimed at improving cognitive control and reducing alcohol-related risk in college populations. Interventions that reduce stress, anxiety, and depressive symptoms may help preserve cognitive flexibility and support more adaptive decision-making in contexts where alcohol use is common (52). Additionally, the interaction between affective distress and alcohol use suggests that individuals experiencing elevated emotional distress may be particularly vulnerable to cognitive inefficiencies when engaging in drinking behavior. This pattern highlights the potential value of integrated prevention approaches that address both emotional regulation and substance use behaviors. While genetic variation in COMT moderated some of these effects, these findings do not support deterministic interpretations of genetic risk. Rather, they suggest that dopaminergic differences may influence sensitivity to environmental and affective conditions relevant to cognitive control.

In contrast to expectations based on prior literature, adverse childhood experiences were not significantly associated with cognitive flexibility in the present sample. However, ACEs were positively correlated with affective distress, consistent with prior research linking early life stress to increased negative affect and internalizing symptoms in emerging adulthood. The lack of direct associations between ACEs and cognitive flexibility in the present study may reflect several possibilities. It is possible that the cognitive effects of early adversity are more strongly expressed through proximal psychological states, such as affective distress, rather than directly impacting executive functioning in young adults. Alternatively, variability in adaptation or resilience following early life stress may reduce the consistency of direct associations with cognitive outcomes in non-clinical samples.

### 4.1 Limitations and future directions

Several limitations should be noted. The cross-sectional design precludes conclusions regarding causality or directionality among affective distress, alcohol use, and cognitive flexibility. The relatively modest sample size may limit power to detect small genetic effects. Furthermore, COMT prevalence is known to vary across different racial and ethnic groups, meaning genotype and ancestry are confounded in these data. Additionally, behavioral measures of cognitive flexibility demonstrated lower reliability relative to self-report measures, consistent with prior work in executive functioning assessment (53). Finally, alcohol use was assessed categorically rather than continuously, which may reduce sensitivity to dose-dependent effects.

An *a priori* power analysis was precluded by the novelty of these complex interactions, particularly given that COMT genotype distributions vary significantly by demographic background, virtually guaranteeing unequal cell sizes. Further, the modest sample size required combining Val/Met and Met/Met participants into a single met-carrier group, consistent with prior studies (41). Although this approach increased power to detect potential genotype effects, this approach limited evaluation of differences among all three COMT genotypes and prevented determination of whether the observed associations were best characterized by an additive, dominant, or recessive genetic model. Consequently, the potential intermediate phenotype associated with heterozygous (Val/Met) carriers could not be examined independently. However, these preliminary data provide a valuable empirical baseline for future power calculations. They also highlight the need for longitudinal designs, such as covariate-adjusted cross-lagged panel models, to resolve the causal ambiguities inherent in the current cross-sectional framework.

## 5 Conclusion

The present findings identify affective distress as a central correlate of cognitive flexibility in college-aged adults, particularly in the context of alcohol use. COMT genotype further moderates these relationships, suggesting that dopaminergic variation may contribute to individual differences in sensitivity to affective and behavioral influences on cognitive control. Together, these results highlight the importance of integrating affective, behavioral, and biological factors when examining cognitive flexibility and alcohol-related risk during emerging adulthood.

## Data Availability

Deidentified data files related to this manuscript can be located at the following OSF repository: https://osf.io/mvwy7/overview?view_only=2511245ecf884bedb81dea1be42139b6.
